# The Influence of Temperature on C153 Steady-State Absorption and Fluorescence Kinetics in Hydrogen Bonding Solvents

**DOI:** 10.1007/s10895-012-1109-2

**Published:** 2012-08-10

**Authors:** Krzysztof Dobek, Jerzy Karolczak

**Affiliations:** 1 Faculty of Physics, Adam Mickiewicz University, Umultowska 85, 61-614 Poznań, Poland; 2 Center For Ultrafast Laser Spectroscopy, Adam Mickiewicz University, Umultowska 85, 61-614 Poznań, Poland

**Keywords:** Coumarin 153, Thermochromism, Solvation, Temperature, Transition dipole moment, Hydrogen bond

## Abstract

In a recent paper (J Fluoresc (2011) 21:1547–1557) a temperature induced modulation of Coumarin 153 (C153) fluorescence lifetime and quantum yield for the probe dissolved in the polar, nonspecifically interacting 1-chloropropane was reported. This modulation was also observed in temperature dependencies of the radiative and nonradiative rates. Here, we show that the modulation is also observed in another 1-chloroalkane—1-chlorohexane, as well as in hydrogen bonding propionitrile, ethanol and trifluoroethanol. Change in the equilibrium distance between *S*_0_ an *S*_1_ potential energies surfaces was identified as the source of this modulation. This change is driven by temperature changes. It leads to a modulation of the fluorescence transition dipole moment and it is the primary source of the experimental effects observed. Additionally, we have found that proticity of the solvent induces a rise in the fluorescence transition dipole moment, which leads to a shortening of the fluorescence lifetime. Hydrogen bonds are formed by C153 also with hydrogen accepting solvents like propionitrile. We show that while such bonds do not affect the transition probability, they do change the S_0_ an S_1_ energy gap which in turn implies a change in non-radiative transition rate in a similar way as in protic solvents, as well as in the fluorescence spectrum position. Finally, the influence of temperature on the energies of hydrogen bonds formed by C153 when acting as hydrogen donor or acceptor is reported.

## Introduction

**Scheme 1:**
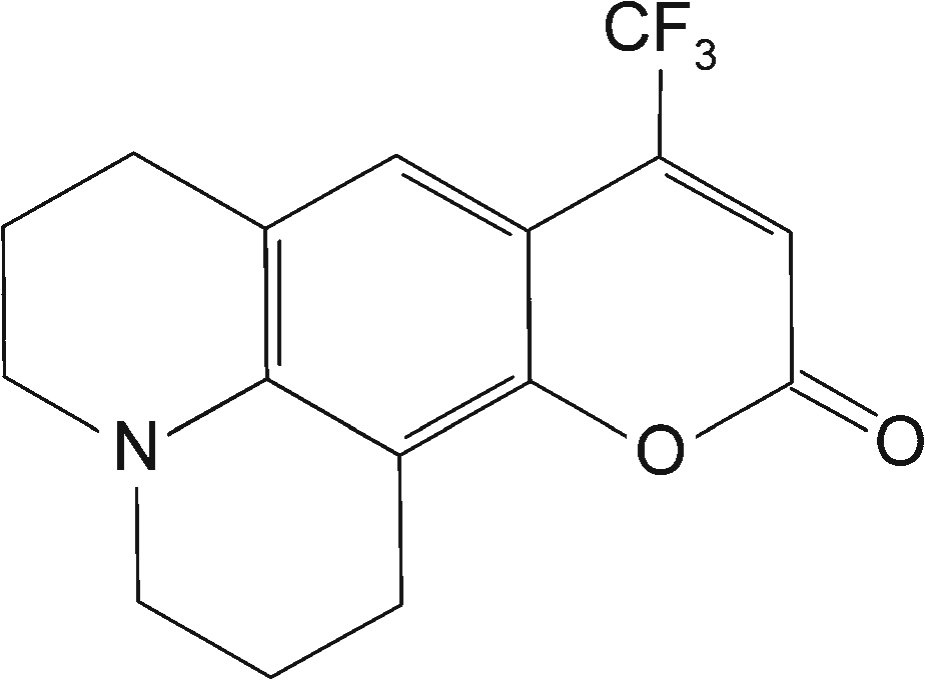
Coumarin 153 (C153) structure

The thermochromism of simple dye molecules is a good indicator of changes in the dye environment polarity following changes in temperature. In many cases thermochromic shifts in absorption and emission reflect the temperature induced changes in refractive index, *n*, and electric permittivity, *ε*, of the solvent as observed by our group in [1,2] for 4-aminophthalimide (4-AP) and Coumarin 153 (C153, Scheme 1) dissolved in several polar non-protic 1-chloroalkanes, or by Suppan et al. in [3–6] for several probes including 4-AP.

However, for a dye interacting specifically with the solvent molecules, temperature influence on the energy of this interaction can affect much more significantly the scale of thermochromic shifts than for a dye showing nonspecific interaction, related to *n*(T) and *ε*(T) dependencies. This additional shift can be of the sign the same as or the opposite to that resulting from nonspecific interactions. In [1,2] we have found both 4-AP and C153 to form hydrogen bonds (H-bond) acting as hydrogen acceptors and donors. Both types of H-bonding interactions result in an additional stabilisation of the first excited singlet state *S*_1_ of both probes. However, in the case of the probes acting as hydrogen acceptors, this additional stabilization has been found to weaken with decreasing temperature. In the case of the dyes acting as hydrogen donors, a slightly rise in the stabilization was observed with decreasing temperature. To identify the origin of such temperature changes in H-bond energies, a preliminary study on the influence of temperature on absorption and emission, including fluorescence time-resolved measurements, was performed for C153 dissolved in 1-chloropropane (ClP) [7]. The purpose of this study was to determine the influence of temperature on the kinetics of C153 deactivation from S_1_ in nonspecifically interacting polar solvents. Surprisingly, C153 fluorescence lifetime (*τ*_F_) and fluorescence quantum yield (ϕ_F_) have been found to follow in this solvent a complex temperature dependence, indicating the intramolecular deactivation is not only controlled by the energy gap law of radiationless deactivation rate.

In this report we would like to present results of our further studies of temperature influence on C153 excitation and deactivation. In order to compare new results with the previous ones obtained for ClP [7], the absorption and emission spectra as well as quantum yield and fluorescence lifetime were measured for C153 dissolved in 1-chlorohexane (ClH) in the temperature range of 183 K–323 K. Additionally, the same spectra and quantities were determined in propionitrile (PPN)—a hydrogen acceptor (Kamlet-Taft polarity scale *α* = 0, *β* = 0.4 [8–10]), in trifluoroethanol (TFEtOH)—a hydrogen donor (*α* = 1.51, *β* = 0) and in ethanol (EtOH)—a hydrogen donor and acceptor (*α* = 0.86, *β* = 0.75). The choice of these solvents was dictated by their H-bonding character and by their melting points, the lower the better.

## Methods

As previously, emission spectra were accumulated using a modified Aminco SPF-500 spectrofluorimeter with single photon counting detection. Absorption spectra were measured using a Jasco V-550 spectrometer. Temperature control was performed using an Oxford Instruments Optistat DN cryostat. Time-resolved fluorescence measurements were made using a TCSPC system with an instrument response function (IRF) of 30 ps full-width at half of the maximum (FWHM). The time per channel was set to 12.2 ps and fluorescence decays were collected into 4,096 channels [11]. A home-made analytical software was used to fit the decays with the simplex approximation algorithm. The signal scattered at excitation light wavelength from a ludox in water solution was used as the IRF. C153 (Fluka) was used as received, 1-chlorohexane (Aldrich), propionitrile (Sigma-Aldrich), ethanol (Sigma-Aldrich) and tri-fluoroethanol (Sigma-Aldrich) were dehydrated using 3 Å (Merck) and 4 Å (Fluka) molecular sieves and the sample were prepared under argon atmosphere after solvent dehydration. C153 concentration was kept at ~10^−5^ M. It is worth underlining the necessity of careful dehydration, whose failure can lead to incorrect results as shown for 4-AP in [12]. In this report we also show results obtained for C153 dissolved in ClH contaminated with water.

## Results

### Steady-State Results

**Fig. 1:**
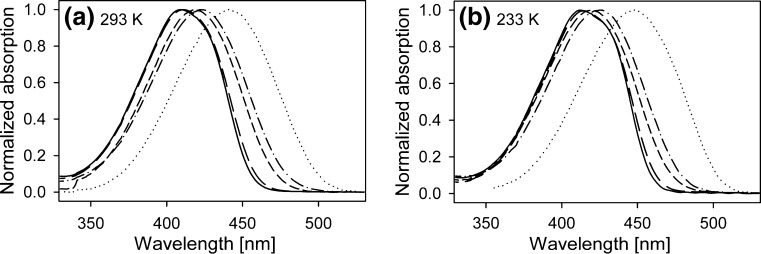
C153 absorption spectra at room temperature (**a**) and at 233 K (**b**) in: ClP (*solid*), ClH (*long dash*), PPN (*short dash*), EtOH (*dot–dash*) and TFEtOH (*dot*)

Steady-state absorption and emission spectra were measured in selected solvents at different temperature ranges with a 10 K step. Figure 1 present normalized absorption spectra of C153 in all solvents used, obtained at room temperature and at 233 K, for the sake of comparison also the ones obtained in 1-chloropropane [7] are shown.

**Fig. 2:**
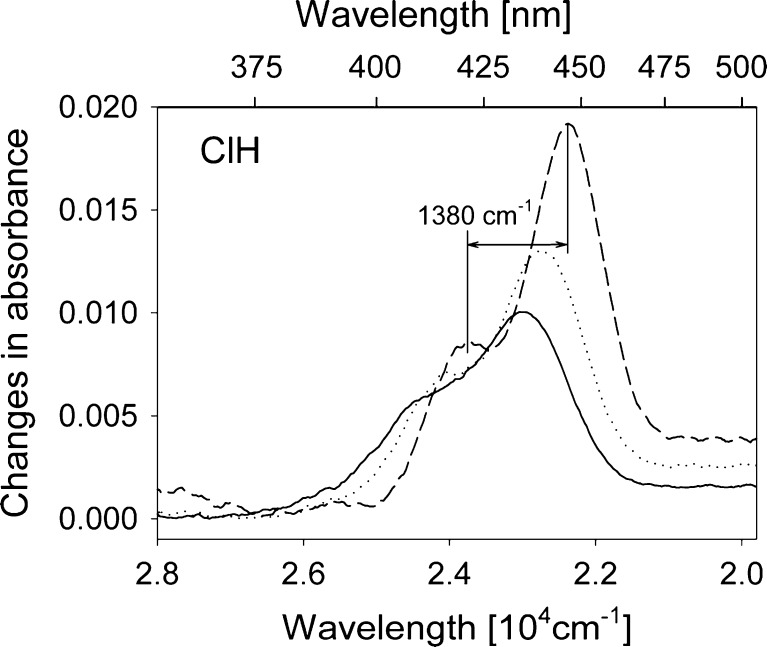
Changes in absorbance of C153 in ClH induced by decrease in temperature: 323 K→313 K (*solid line*), 263 K→253 K (*dot*) and 193 K→183 K (*dash*)

A subtle difference in the shape of the spectra recorded at the two temperatures can be noted for ClP and ClH. To better resolve this change it is convenient to determine the differences in absorbance at consecutive temperatures at which measurements were made, separated by 10 K. Figure 2 presents such “derivatives” for C153 in ClH.

Note that the curves shown in Fig. 2 include the effects resulting from solvatochromic shifts of absorption, thus they cannot be directly interpreted as a result of temperature induced intramolecular changes. Nevertheless, in ClH (and ClP, not shown) a vibronic structure of the changes in absorbance can be seen. In Fig. 2 the distance in energy of 1,380 cm^−1^ is drawn separating two consecutive maxima of the curve for the lowest temperature change. This value is close to the 1,150 cm^−1^ frequency of the deactivation accepting mode found in the emission in ClP [7] and in gas-phase [13,14]. In other solvents no structure can be observed which would well correspond to the lack of any clear change in absorption spectra shape when decreasing temperature. Note that the ratio of the amplitude of two maxima visible in each curve in Fig. 2 changes with decreasing T in favour of the highest most red shifted peak.

**Fig. 3:**
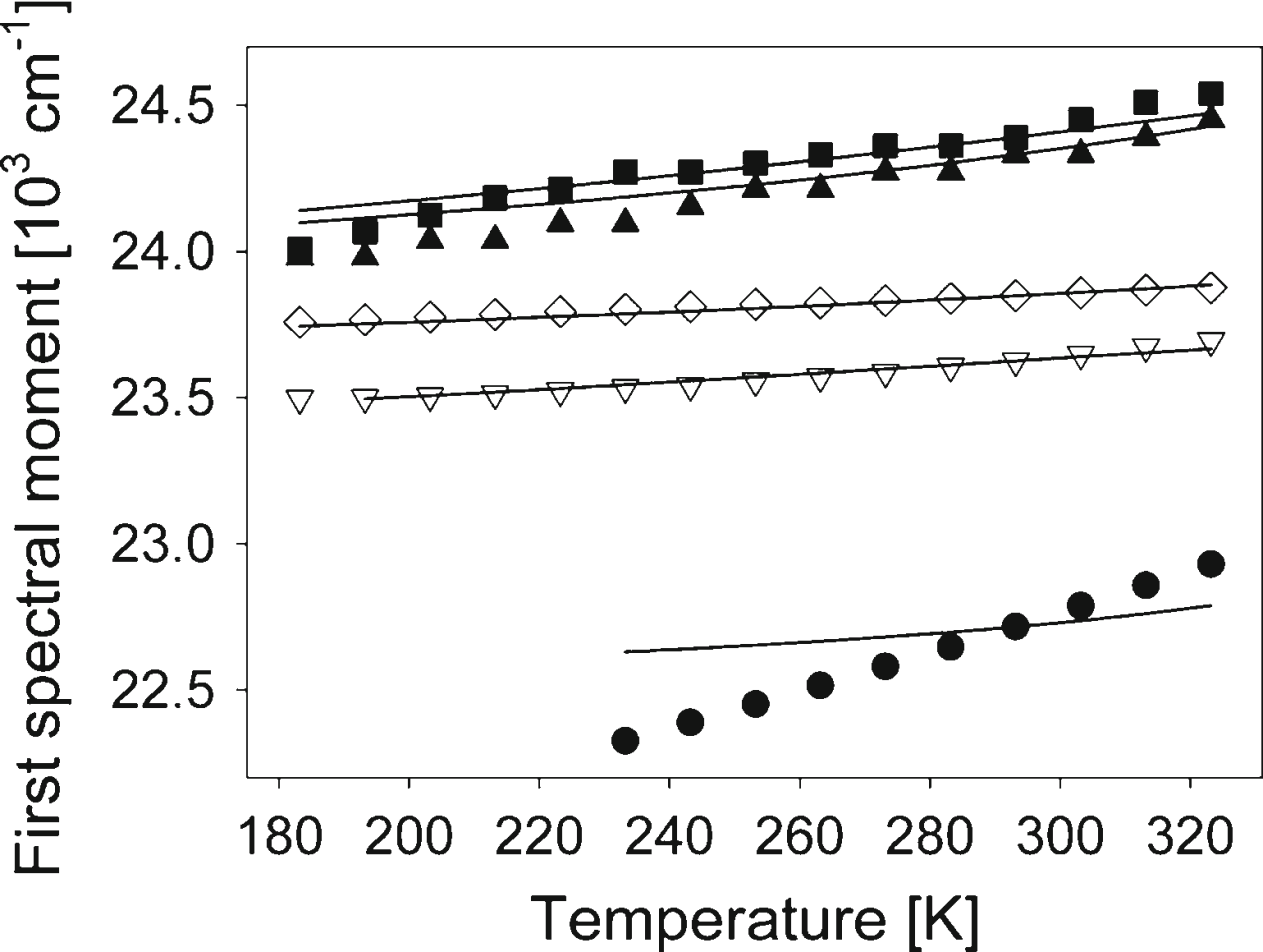
C153 steady-state absorption spectra maximum positions (*ν*_p_) vs temperature in: ClH (*filled squares*), ClP (*filled triangles*), PPN (*empty diamonds*), EtOH (*empty triangles*) and TFEtOH (*filled circles*). *Lines* correspond to theoretically determined positions

Figure 3 shows the temperature dependence of the peak positions, *ν*_p_, of absorption spectrum S_0_→S_1_ band in all five solvents. Lines represent *ν*_p_(T) slopes predicted theoretically in the way described later in the text.

In PPN, EtOH and TFEtOH the peak positions correspond to *ν*_p_ of LogNormal curves fitted to the absorption spectra. For ClP and ClH, due to the vibronic structure of the spectra, no correct fit could be obtained by means of LogNormal functions. Thus, in this case *ν*_p_ corresponds to frequencies at which the maxima of the consecutive absorption spectra were observed. The difference in approaches results in a smoother T dependence in the three specifically interacting solvents.

**Fig. 4:**
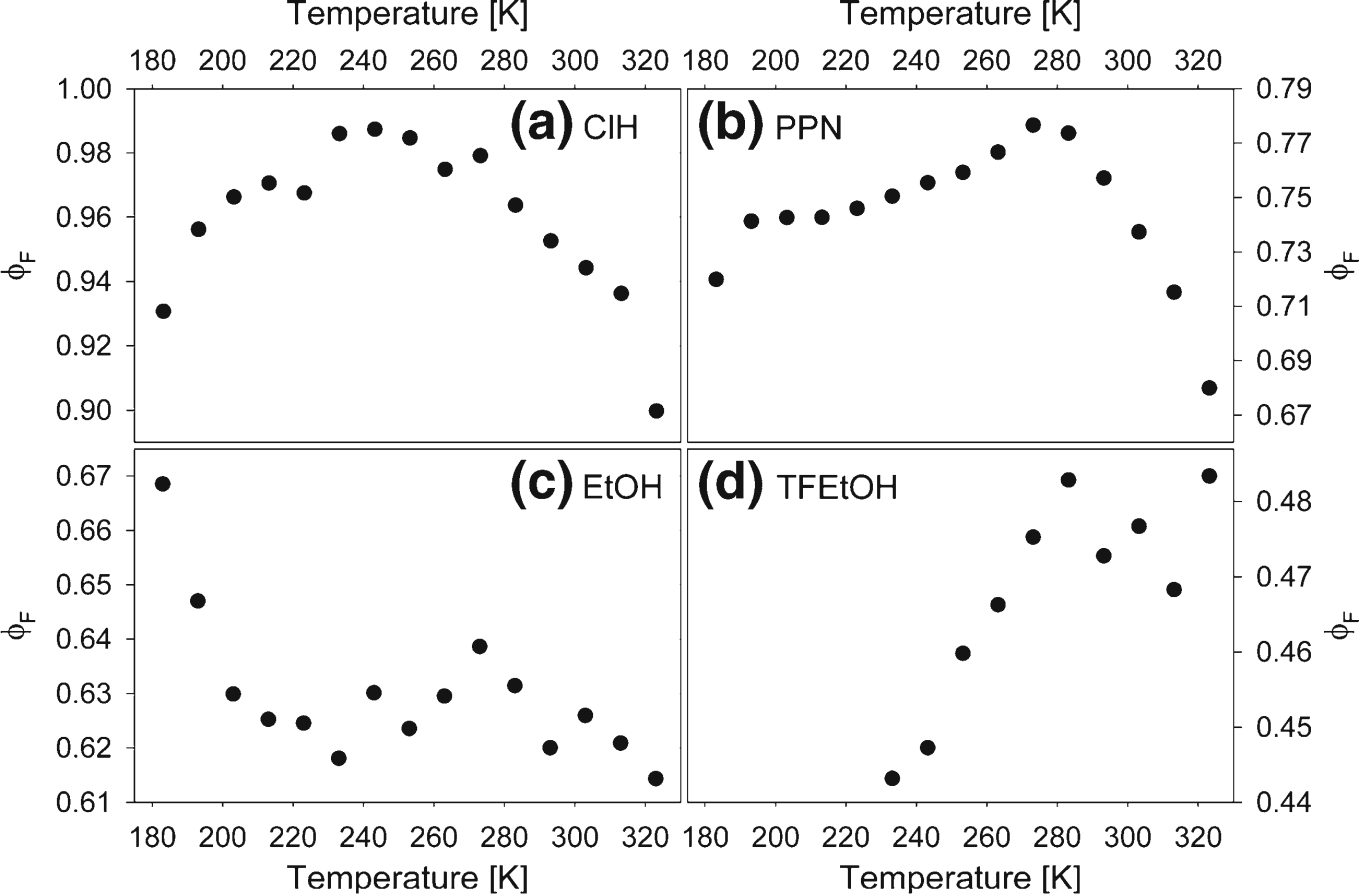
Temperature dependencies of the fluorescence quantum yield, ϕ_F_, of C153 in ClH (**a**), PPN (**b**), EtOH (**c**) and TFEtOH (**d**)

Using quinine sulphate in 0.05 M H_2_SO_4_ (ϕ_F_ = 0.52) as a standard, C153 fluorescence quantum yield, ϕ_F_, in all solvents was determined at room temperature. Then, by assuming the room temperature emission spectrum in selected solvent as a standard, temperature dependencies of ϕ_F_ were determined, shown in Fig. 4.

Values of ϕ_F_(T) follow similar dependencies in ClH and PPN, while in EtOH and TFEtOH they are significantly different. Note, that ϕ_F_(T) in TFEtOH is quite similar to the one found in ClP [7]. Only in EtOH ϕ_F_ rises at the lowest temperatures. Analysis of time-resolved emission spectra (TRES), discussed in the next section, have shown that this rise is a consequence of a significant retardation of solvation dynamics in EtOH at low temperatures. In all solvents ϕ_F_(T) slope changes from negative into positive at distinct temperatures: 243 K in ClH, 273 K in PPN, 273 K in EtOH and 283 K in TFEtOH. In ClP it was 273 K. The changes observed in ϕ_F_ are twice as high in ClH and PPN as those in EtOH and TFEtOH.

### Time-Resolved Results

**Fig. 5:**
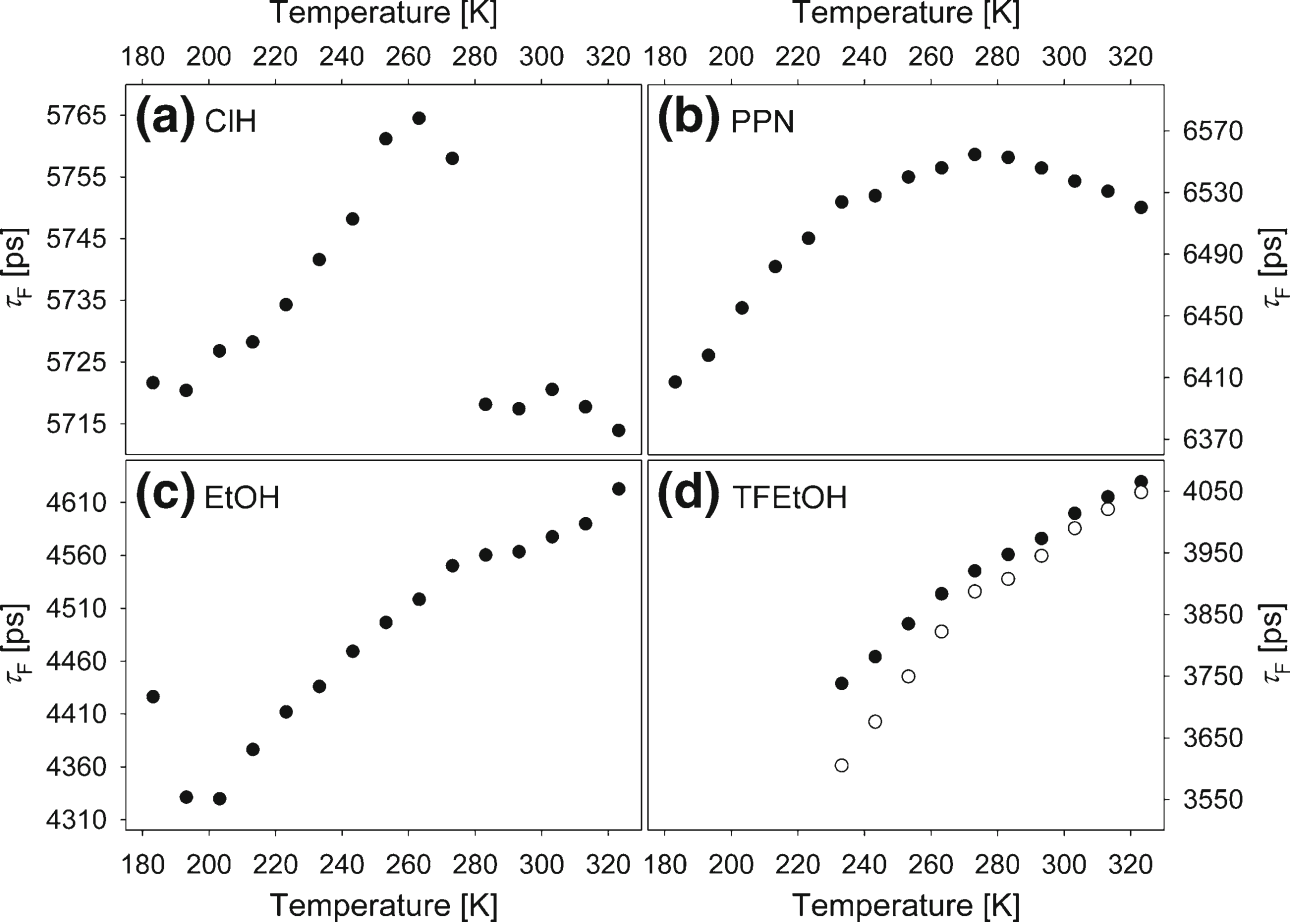
Temperature dependencies of the fluorescence decay time for C153 in ClH (**a**), PPN (**b**), EtOH (**c**) and TFEtOH (**d**). In the alcohols the dominant long component time (*filled circles*) of the double-exponential decay function fitted to the experimental decays is presented along with the single exponential decay time for TFEtOH (*empty circles*)

First of all, fluorescence decays were measured for C153 in all solvents in the same temperature ranges as in the case of steady-state absorption and emission spectra. Due to some limitation of our experimental setup, during the experiment the excitation wavelength was constant and set to the maximum of the room temperature absorption spectrum in a selected solvent, while the emission wavelength was set to the maximum of the emission spectrum at a selected temperature. In ClH and PPN the decay was found to be properly described by a single exponential decay function in the full temperature range. In EtOH below 293 K and in TFEtOH in the full T range a double exponential fit was necessary to correctly reconstruct the data. The double exponential function consisted of a dominant long component. A decrease in temperature induced a change in the second component decay time from 100 ps (contribution F = 0.2 %) to 2,200 ps (F = 25 %) in EtOH, and from 100 ps (F = 0.6 %) to 900 ps (F = 4 %) in TFEtOH. The long component decay time had been found in both protic solvents to follow a similar temperature dependence as the single exponential decay time, also fitted to the data. Therefore, Fig. 5 shows *τ*_F_ of the single exponential fit for ClH, PPN and the long component of the double exponential fit for both protic solvents.

There is a similarity in *τ*_F_(T) dependencies in ClH and PPN, as well as in EtOH and TFEtOH. Similarly, as for ϕ_F_(T) there are characteristic temperatures at which changes in *τ*_F_(T) slope are observed in ClH (263 K) and PPN (273 K). In alcohols a modulation of *τ*_F_(T) can be observed in T ranges between 300 K and 273 K, the same in which changes in ϕ_F_(T) slope occur. The scale of the changes in *τ*_F_ seems to be linked to the polarity of the solvent, the higher the polarity the larger the range of the *τ*_F_ values. This observation includes the results for C153 in ClP from [7].

**Fig. 6:**
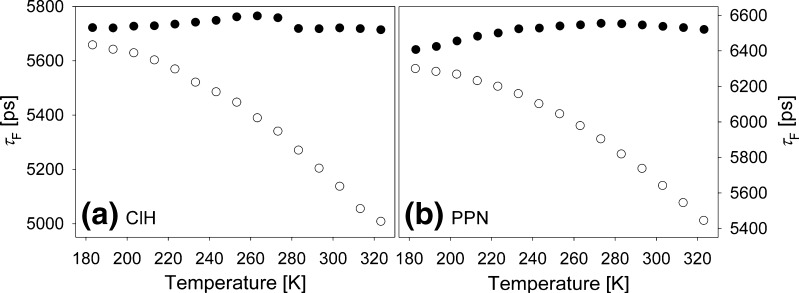
Temperature dependence of fluorescence decay time of C153 in dehydrated ClH (**a**)–*filled circles*, in ClH+water mixture at 2.2⋅10^−3^ M water concentration (**a**)–*empty circles*, in dehydrated PPN (**b**)–*filled circles* and in improperly dehydrated PPN (**b**)–*empty circles*

Effects of improper dehydration of 1-chloroalkanes on 4-AP thermochromism were reported in Ref. [12]. For C153 in ClP and ClH we also noticed that incorrect dehydration of these solvents led to the *τ*_F_(T) dependencies significantly different from that shown in Fig. 5 and in Ref. [7]. A similar observation was also made for C153 in PPN. Thus, following the procedure undertaken in Ref. [12] we had preliminarily dehydrated 50 mL of ClH and next we added to it 2 *μ*L of distilled water, which corresponded to a 2.2⋅10^−3^ M water concentration. Fluorescence decays in such a ClH+water mixture were measured at the wavelengths corresponding to the maxima of steady-state spectra collected at subsequent temperatures. They were found to be described by double exponential decay functions with a dominant 5–6 ns component and a minor ~200 ps second component whose contribution did not exceed 1 %. Figure 6a presents the temperature dependence of the long component for C153 dissolved in ClH+water mixture along with the dependence found in dehydrated ClH shown already in Fig. 5. Additionally, Fig. 6b shows the impact of improper dehydration of PPN on C153 *τ*_F_(T) dependence. The properly dehydrated sample (filled circles) was prepared with PPN dried with molecular sieves twice as long as for the improperly dehydrated one (empty circles), after changing the molecular sieves once. It was also prepared under argon atmosphere in contrast to the sample still contaminated by water.

Water presence makes fluorescence decays much shorter at high temperatures. Lowering temperature induces *τ*_F_ rise, which asymptotically approaches the values found in dehydrated solvents at the lowest temperatures. These results, combined with *τ*_F_ values obtained for C153 in EtOH and TFEtOH, show that the interaction of C153 with protic solvents leads to a shortening of *τ*_F_. To check if C153+water complexes are formed only in the ground state or also C153+water exciplexes are formed after excitation, fluorescence decays were measured in ClH+water solution at three different wavelengths and at 323 K, 293 K and 263 K. The wavelengths were selected from the blue and red ranges of the steady-state spectrum and at its maximum. At the fourth temperature of 213 K fluorescence decays were measured at seven different wavelengths covering the full spectrum. Multi-exponential decay functions were fitted to these experimental decays. None from among the fitted functions had a component bringing a significant negative contribution. This indicates that water forms complexes with C153 only in the ground state of the probe, in contrast to 4-AP dissolved in the same solvent mixture [12]. The same is assumed to be true in PPN. Such a conclusion is justified as C153 lifetime is three times shorter than that of 4-AP, while water association is much more effective in the latter dye. However, in contrast to 4-AP for which two decay components were found in ClH+water mixture; one corresponding to free 4-AP and the other to 4-AP-water complex/exciplex, C153 decay is described mainly by a single exponential component which must correspond to the C153+water complex. Table 1 gives the fluorescence decay components times and amplitudes found at selected wavelengths.

**Table 1:** Times and amplitudes of fluorescence decay components found for C153 in ClH+water mixture at 2.2 × 10^−3^ M water concentration and at selected temperatures and wavelengths

T [K]	*λ* [nm]	*τ*_1_ [ps]	a_1_	*τ*_2_ [ps]	a_2_
323	445	4494	0.82	130	0.18
	483	5008	0.93	190	0.07
	556	5011	0.98	281	0.02
293	450	5152	0.75	87	0.25
	492	5204	0.94	207	0.06
	560	5221	1.07	16	−0.07
263	450	5397	0.88	2045	0.12
	490	5390	0.90	131	0.10
	563	5462	0.98	170	0.02
213	445	5466	0.36	131	0.64
	490	5603	0.90	217	0.10
	560	5609	1.38	50	−0.38

Increase in *τ*_1_ with fluorescence wavelength reflects solvation of ClH and/or preferential solvation of water. Lack of rise in *τ*_2_ with decreasing T indicates this component is not related to free C153, but is also a manifestation of solvation dynamics.

The multi-exponential character of the fluorescence decays observed for C153 in EtOH and TFEtOH is similarly as the ϕ(T) dependence in EtOH, connected to significantly slower solvation dynamics of the probe in alcohols than in the two other solvents. Such a conclusion follows from analysis of TRES determined for C153 in EtOH at 293 K, 233 K and 193 K and in TFEtOH at 293 K and 233 K. TRES were determined in a usual way. First, fluorescence decays at 12 selected wavelengths with a 15 nm step were determined in EtOH, starting from 480 nm, and in TFEtOH starting from 490 nm. The experimental signals were fitted by multi-exponential decay functions. Next, the decay functions resulting from the fit were normalized in such a way as to equalize the area under the decay to the steady-state fluorescence spectrum intensity at selected wavelength and temperature. Steady-state spectra were corrected for the detector sensitivity and scaled by the *λ* ^2^ factor. From the normalized decays TRES were reconstructed. Next, each spectrum of the TRES corresponding to subsequent time delay was fitted by a LogNormal function, with the amplitude, asymmetry, peak frequency *ν*_p_ and half-width as the parameters of the fit. From *ν*_p_ values the correlation function $C(t) = \left( {{v_p}(t) - {v_p}\left( \infty \right)} \right)/\left( {{v_p}(0) - {v_p}\left( \infty \right)} \right)$ was determined. Here, *ν*_p_(∞) corresponds to the peak frequency of the LogNormal curve fitted to the TRES spectrum at the longest time delay. Finally, C(t) dependencies were fitted by single- and double-exponential decays, depending on which number of exponential components was found necessary to reproduce C(t) properly. Table 2 presents the obtained C(t) decay time values, *τ*_si_, along with the component amplitudes, b_i_, and the average decay time defined as $\left\langle {{{\tau }_{s}}} \right\rangle = \left( {\sum\limits_{i} {{{b}_{i}}{{\tau }_{{si}}}} } \right)/\sum\limits_{i} {{{b}_{i}}}$.

**Table 2:** Decay time values, *τ*_si_, and amplitudes, b_i_, of the exponential decay components fitted to the solvation correlation function, C(t), determined for C153 in EtOH and TFEtOH at selected temperatures. 〈*τ*_s_〉 – average decay time

Solvent	T [K]	*τ*_s1_ [ps]	b_1_	*τ*_s2_ [ps]	b_2_	〈*τ*_s_〉 [ps]
EtOH	293	35	1	–	–	35
	233	70	0.45	228	0.55	157
	193	208	0.35	974	0.65	704
TFEtOH	293	77	0.66	257	0.34	138
	233	50	0.23	378	0.77	302

The values reported in Table 2 describe only the slowest part of C(t) decays, because of a small time-resolution used here. There is a good agreement between the value obtained for C153 in EtOH at 293 K and the longest C(t) component reported in [15]. The solvation dynamics is slower in TFEtOH at room T. However, if we analyze the relative change in ⟨*τ*_s_⟩ in EtOH and TFEtOH with decreasing T, we see that the solvation is slowed by decreasing T much more efficiently in EtOH. Additionally, this retardation grows quickly at the lowest temperatures in this solvent. This is the reason why ϕ_F_ in Fig. 4 and *τ*_F_ in Fig. 5 increase in EtOH at the lowest T. Strongly retarded solvation at this T range prevents the steady-state spectrum from shifting fully to the red. As a result, fluorescence deactivation rate (*k*_*F*_) proportional to *ν* ^3^ as well as to ϕ_F_, increases. Additionally, ϕ_F_ which is calculated by integration of the fluorescence steady-state spectrum, rises because the integration range is shifted to the blue, thus to higher energies. Finally, as mentioned earlier, the retarded solvation is responsible for the bi-exponential character of the fluorescence decays in the alcohols measured at the maximum of the steady-state fluorescence spectra. We assume in the next section that the dominant longest component of the fluorescence decays in EtOH and TFEtOH bring reliable information about C153 deactivation rate, at least in the range of high temperatures. However, for the lowest T such assumption is clearly invalid, thus the interpretation of the kinetic results presented below is correct for C153 in alcohols only in the high temperature range.

## Discussion

Thermochromic shifts of C153 emission spectra reported in [2] indicated that the probe forms H-bond with hydrogen accepting solvents, and that the change in energy of this bond, stimulated by probe excitation, increases with decreasing temperature, at least in the vibrationally and solvent relaxed first singlet excited state, $S_1^{rel}$. These results were obtained as a difference between the emission spectrum positions predicted by the Onsager model and the experimental position values. To evaluate in a similar way the possible influence of specific interactions on absorption peak positions the Onsager model was applied using Eq. 1 [2,16].

1 $$ \matrix{ {\Delta {v_{Abs}} = 2\frac{{{\mu_g}\left( {{\mu_g} - {\mu_e}} \right)}}{{{a^3}}} \cdot \left( {\frac{{\varepsilon - 1}}{{\varepsilon + 2}} - \frac{{{n^2} - 1}}{{{n^2} + 2}}} \right)} \\ { + \frac{{\mu_g^2 - \mu_e^2}}{{{a^3}}} \cdot \left( {\frac{{{n^2} - 1}}{{2{n^2} + 2}}} \right)} \\ { + \frac{{6\mu_g^2\left( {{a_g} - {a_e}} \right)}}{{{a^6}}} \cdot {{\left( {\frac{{\varepsilon - 1}}{{\varepsilon + 2}} - \frac{{{n^2} - 1}}{{{n^2} + 2}}} \right)}^2}} \\ { + \frac{{3\left( {{a_g} - {a_e}} \right)J{J_S}}}{{2hc{a^3}\left( {J + {J_S}} \right)}} \cdot \frac{{{n^2} - 1}}{{{n^2} + 2}}} \\ } $$

**Fig. 7:**
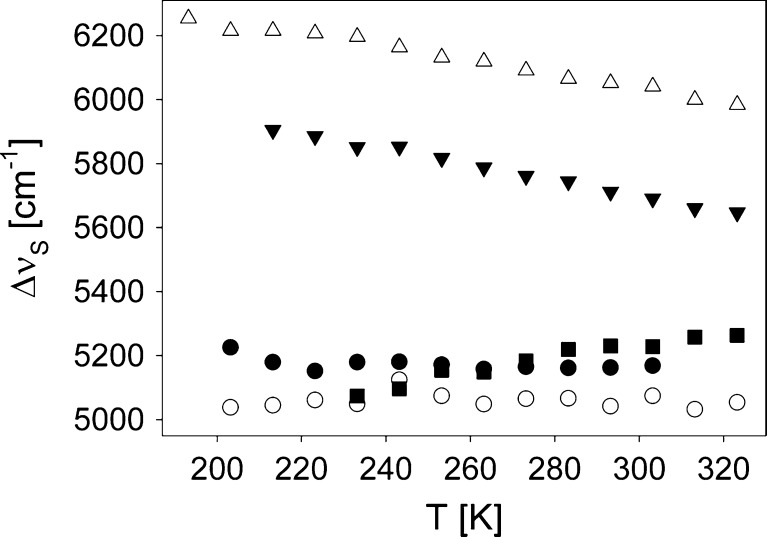
Stokes shift vs temperature for C153 in: ClP (*filled circles*), ClH (*empty circles*), PPN (*filled triangles*), EtOH (*empty triangles*) and TFEtOH (*filled squares*)

Above, *μ*_g_ and *μ*_e_ are C153 dipole moments in the ground and excited states, *a* is the Onsager radius of the probe molecule, *α*_*g*_ and *α*_e_ are the ground and excited state probe polarizabilities, *J* and *J*_S_ are the probe and solvent ionization potentials and *h* and *c* have the usual meanings. These calculations were made assuming the same parameter values for C153 in the Franck-Condon $\left( {S_0^{FC},\,S_1^{FC}} \right)$ and in the relaxed $\left( {S_0^{rel},\,S_1^{rel}} \right)$ states, thus assuming the same dipole moment *μ*_*g*_ = 6.6 D [17] in $S_0^{FC}$ and $S_0^{rel}$, *μ*_*e*_ = 9.7 D in $S_1^{FC}$ and $S_1^{rel}$, and the same *α*_*g*_ in both *S*_0_ states and *α*_*e*_ in both *S*_1_ states [2]. EtOH ionization potential *J*_S_ = 11.05 eV was determined using AM1 hamiltonian with the MOPAC suite, EtOH *n*(*T*) and *ε*(T) were determined from the data given in [9]. For the other C153 and solvents parameters see [1,2]. The shifts in absorption of C153 in all solvents were predicted at subsequent temperatures. The values of ∆*v*_Abs_ were compared with experimental *ν*_p_(T) in the following way: for a selected solvent the absorption spectrum peak value *ν*_p_(293 K) was added to the absolute value of the shift ∆*v*_Abs_ (293 K). In this way a pseudo “gas-phase” $\nu_p^{g\prime \prime }$ position of the spectrum was obtained. Next, from $\nu_p^{g\prime \prime }$ the absolute values of ∆*v*_Abs_(T) were subtracted at subsequent temperatures different from 293 K. The results of such a procedure are shown in Fig. 3 as solid lines. It led to the following $\nu_p^{g\prime \prime }$ values: 26,060 cm^−1^ (ClH), 26170 (ClP), 26120 (PPN), 25840 (EtOH) and 24920 (TFEtOH). As expected, $\nu_p^{g\prime \prime }$ values are not the same as they were determined assuming that all solvents interacted exclusively nonspecifically which is obviously incorrect. But, they indicate which solvents most probably interact specifically with C153 in the ground state and these solvents are EtOH and TFEtOH. It is especially important that $\nu_p^{g\prime \prime }$ in PPN fall into the same range as found for 1-chloroalkanes, and that the *v*_*p*_ (T) dependence slope in PPN corresponds exactly to the ∆*v*_Abs_ (T) dependence slope, see Fig. 3. Both these observations indicate that PPN interacts only nonspecifically with C153 in $S_0^{rel}$, or that the H-bond formed by PPN with C153 do not change in energy after C153 excitation to $S_1^{FC}$, contrary to what was deduced from the emissive results for C153 $S_1^{rel}$ in the same solvent [2]. On the other hand, a comparison of $\nu_p^{g\prime \prime }$ values obtained in 1-chloroalkanes and PPN with those found in alcohols indicates that in EtOH a slight additional specific stabilisation take place already in $S_1^{FC}$, while in TFEtOH the specific interaction with C153 in $S_1^{FC}$ is significantly higher in energy than in $S_0^{rel}$. No temperature dependence of this additional specific stabilisation is observed in EtOH, while in TFEtOH its energy increases with decreasing temperature. This last result is in contrast to that found for $S_1^{rel}$ [2]. It means that the Stokes shift in this solvent should decrease with decreasing temperature and indeed it is observed. Figure 7 shows the temperature dependence of the experimental Stokes shifts, ∆*v*_S_, for C153 in all solvents.

**Fig. 8:**
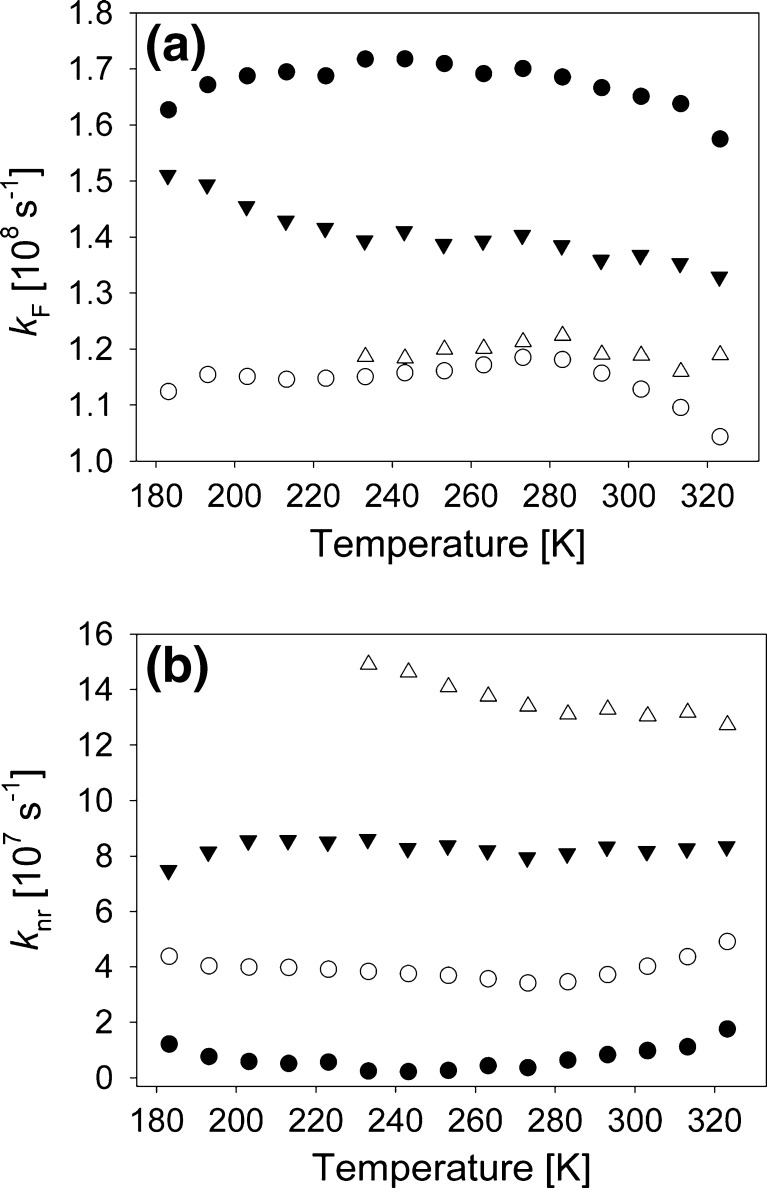
Temperature dependencies of the radiative, (**a**) *k*_F_, and non-radiative, (**b**) *k*_nr_, rates in ClH (*filled circles*), PPN (*empty circles*), EtOH (*filled triangles*) and TFEtOH (*empty triangles*)

The independence of ∆*v*_*S*_ of temperature in 1-chloroalkanes is unexpected as the emitting $S_1^{rel}$ state is more strongly stabilized than $S_1^{FC}$ due to the reorientational solvation of the solvent taking place when C153 is relaxed. The energy of this additional stabilizing interaction should increase with decreasing temperature, which in turn should lead to an increase in ∆*v*_S_. However, the Onsager model (Eqs. 1 and 2 in [2]) reveal that the theoretical Stokes shift in ClP and ClH changes only by 90 cm^-1^ within the 120 K temperature interval studied in this work—a value in the range of the error of the points in Fig. 7 for C153 in 1-chloroalkanes. In PPN, EtOH and TFEtOH even a smaller ∆*v*_S_ change is expected from the Onsager model. In contrast, clear ∆*v*_S_ (T) dependencies in these solvents are observed. In TFEtOH ∆*v*_S_(T) dependence reflects the above-described effects. In PPN and EtOH the increase in ∆*v*_S_ must be related to an additional stabilization of C153 in $S_1^{rel}$ state, rising in energy with decreasing temperature. This conclusion is in accordance with the results shown in [2]. The difference between the expected positions of the C153 emission spectra in methanol, PPN and TFEtOH and the experimental ones have been found to show temperature dependencies with the same slope signs as observed in Fig. 7 for ∆*v*_S_ in the same two solvents. The analysis of the results given in [2] and in this work is based on the continuum solvent Onsager model, thus their reliability can be questioned by those who believe that this model fails in solvation description. Additionally, we have found the absorption spectrum full width at half of the maximum (fwhm) to be higher than that of the emission spectrum in all solvents, at each temperature. According to [18] such an observation can be a manifestation of a nonlinear dependence of the local solute potential on the solute dipole moment. This observation would however need a much deeper study, as absorption and emission spectra fwhm are strongly dependent on the frequency of the most active vibrational mode in a selected type of transition. Our results, shown later, and these presented in [15] reveal this frequency to be higher in the absorbing ground state, $S_0^{rel}$, than in the emitting $S_1^{rel}$. Nevertheless, the experimental ∆*v*_S_(T) dependence obtained for C153 in PPN compared to that found in 1-chloroalkanes gives a very strong argument for the presence of specific interactions between C153 in $S_1^{rel}$ and PPN molecules. Thus, the results show that C153 can act as a hydrogen donor. The presence of this type of H-bonding, however, does not influence C153 *τ*_F_(T) dependence, as can be deduced from results presented in Fig. 5. These results show that the protic character of the solvent has a significant impact on the *τ*_F_(T) dependence, when compared to non-protic solvents (ClP as well [7]). Overall, the shortest lifetime is observed in the most protic TFEtOH, longer in EtOH, ClH, and the longest in PPN. In alcohols a slight modulation of the linear *τ*_F_(T) dependence can be observed, while in ClH and PPN the values of *τ*_F_ changes with temperature in a similar way as in ClP, that is the slope of *τ*_F_(T) changes in sign at a temperature in the range 260÷280 K. In both protic solvents a small modulation of *τ*_F_(T) takes place in the same temperature range in which the change in sign of the *τ*_F_(T) dependence occurs in ClH and PPN. Thus, we can assume that H-bonding with protic solvents dilutes C153 *τ*_F_(T) dependence resulting from pure intramolecular deactivation observed in ClH and PPN. Non-negligible is also the retarded solvation, which however as shown for C153 in EtOH is expected to lead to an increase in *τ*_F_. The *τ*_F_(T) dependence in neat solvents cover a narrow range of *τ*_F_ values as can be seen in Fig. 6. Such subtle changes in *τ*_F_ are also a manifestation of the narrow ranges in which ϕ_F_ changes in all solvents. Together these quantities gives the radiative, *k*_F_, and non-radiative, *k*_nr_, rates. Figure 8 show their temperature dependencies for all four solvents.

**Fig. 9:**
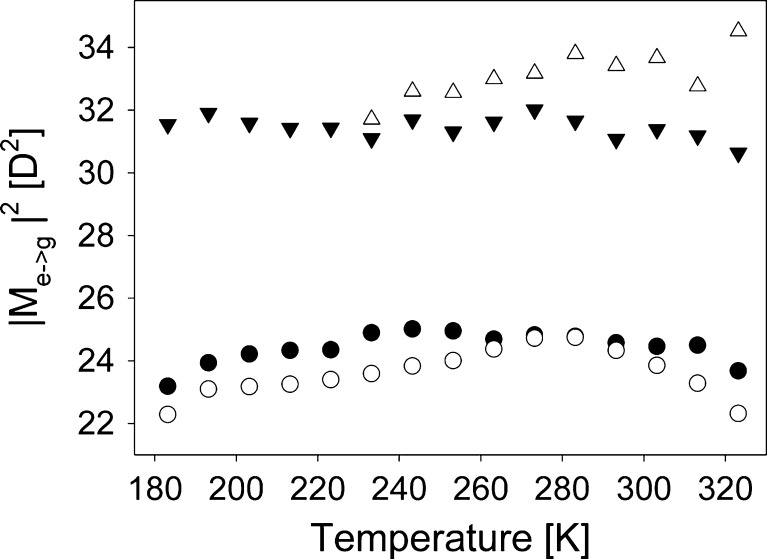
Modulus squared of fluorescence (M_e→g_) transition dipole moments in ClH (*filled circles*), PPN (*empty circles*), EtOH (*filled triangles*) and TFEtOH (*empty triangles*)

Non-radiative deactivation in C153 is known to be governed by internal conversion. According to our knowledge no ISC for this molecule has ever been observed. Relative *k*_nr_ values at the same temperatures in all solvents indicate that the energy-gap law partly controls non-radiative deactivation. The order in which *k*_nr_ values increase at a selected temperature corresponds well to the energy of emission in the solvents, thus to the emission position. However, the energy-gap law predicts an exponential decay of *k*_nr_ with temperature rising [19,20]. The temperature range in which measurements were made does not correspond to the tail of decaying *k*_nr_(T) dependence, see Fig. 8 in [7]. In EtOH the non-exponentiality is evident in the low temperature range. However, in this solvent the retardation of the solvent relaxation is the source of the low-temperature *k*_nr_(T) dependence, as the emission spectrum is not shifted totally to the red, which in turn leads to a drop in *k*_nr_ at the lowest T. In ClH and PPN *k*_nr_(T) slope sign changes at higher temperatures. Similarly to what was observed for C153 in ClP [7], this effect is in conflict with the energy-gap law. Radiative rate *k*_F_(T) dependencies have also similar features in all solvents. Using them, the modulus squared emission transition dipole moments at subsequent temperatures were determined, as shown in Fig. 9. These values were found using the Birks equation [21]:

2 $$ \left| M \right|_{e \to g}^2 = \frac{{3h}}{{64{\pi^4}}}\frac{1}{{{n^3}}}{k_F}\widetilde{v}_F^{ - 3}, $$

3 $$ \widetilde{v}_F^{ - 3} = \frac{{\int {I(v){v^{ - 3}}dv} }}{{\int {I(v)dv} }}. $$

Now, we see that the $S_1^{rel} \to S_0^{FC}$ radiative transition probability differs in protic solvents from that in non-protic ones. Once again we stress that the results at lower temperatures in alcohols (especially in EtOH) are not reliable. Except for PPN, the T dependencies in the other solvents are not as well defined as for *τ*_F_. It is connected to the noise in ϕ_F_ and to the range of values in which *τ*_F_ changes in a particular solvent. We would like to point out that *τ*_F_(T) results are blurred by a much smaller relative noise, comparing to ϕ_F_(T), which was determined indirectly from the results obtained by two different instruments. However, in ClH similarly as in PPN, we can see that M_e→g_ starts to rise with T increasing from 183 K to end by falling at the highest temperature. It is also interesting to notice that except for the points at 323 K, M_e→g_ follows similar changes (increase/decrease) in both protic solvents. It suggests that an additional modulation of M_e→g_ may be present in both solvents, however it is out of reach of the resolution of our equipment. Changes in M_e→g_ indicate changes in the equilibrium distance between C153 *S*_0_ and *S*_1_ potential energy surfaces. To check whether steady-state emission of C153 in PPN and ClH reflects such changes, the same model of emission spectrum was applied as in [7], described in [22,23]. According to this model, the emission spectrum of the molecule in a homogeneous non-interacting solvent can be defined as (MKS):

4 $$ I(v) = \sum\limits_{{n_1}} {\sum\limits_{{n_2}} { \ldots \sum\limits_{{n_N}} {{I_{{n_1}{n_2} \ldots {n_N}}}} } } (v) $$

5 $$ {I_{{n_1}{n_2} \ldots {n_{\text{N}}}}}\left( \nu \right) = {\left( {\frac{{{E_{00}} - \sum\limits_{{\text{j}} = 1}^{\text{N}} {{n_{\text{j}}}h{\nu_{\text{j}}}} }}{{{E_{00}}}}} \right)^3}\prod\limits_{{\text{j}} = 1}^{\text{N}} {\left( {\frac{{\xi_{\text{j}}^{{n_{\text{j}}}}}}{{{n_{\text{j}}}!}}} \right)} \exp \left[ { - 4\left( {\ln (2)} \right){{\left( {\frac{{h\nu - {E_{00}} + \sum\limits_{{\text{j}} = 1}^{\text{N}} {{n_{\text{j}}}h{\nu_{\text{j}}}} }}{{\Delta {\nu_{1/2}}}}} \right)}^2}} \right] $$

6 $$ {\xi_j} = {{1} \left/ {2} \right.}{\left( {{\Delta_j}} \right)^2} $$

Emission intensity is given in units of the number of quanta emitted per energy interval [23]. The number of modes is truncated to N most active accepting vibrational modes in the ground state. *E*_00_ is the energy gap between the lowest levels of the fitted vibronic modes of *S*_0_ and *S*_1_ states. The parameter *n*_j_ is the vibronic number of the j-th accepting mode. The summations in Eqs. (2, 3) has been limited to N = 1, *n*_1_ = 6. The symbol ν_j_ is the j-th accepting mode frequency. As the modes number was set to unity, we use below a substitution j=A, while *ν* is the frequency at which the emission spectrum is measured, $\Delta {\overline v_{1/2}}$ is the full width at half-maximum of the Gaussian inhomogeneous broadening, and ${\overline \xi_{\text{A}}}$ is the Huang-Rhys factor related to the equilibrium bond distance difference, ${\overline \Delta_{\text{A}}}$, between the excited and ground states, that is the parameter we are interested in. The function given in Eq. (2) served as a fitting model of the emission spectra at subsequent temperatures. *E*_00_, ${\overline v_{\text{A}}}$, ${\overline \xi_{\text{A}}}$, $\Delta {\overline v_{1/2}}$ and a scaling *a*_0_ factor (by which *I*(*ν*) was multiplied) were parameters of the fit. The overlines in the symbols indicates ${\overline v_{\text{A}}}$ is in fact an average mode. The quality of the fit was estimated using the *χ* ^2^ value and the errors of the fitted parameters values.

**Fig. 10:**
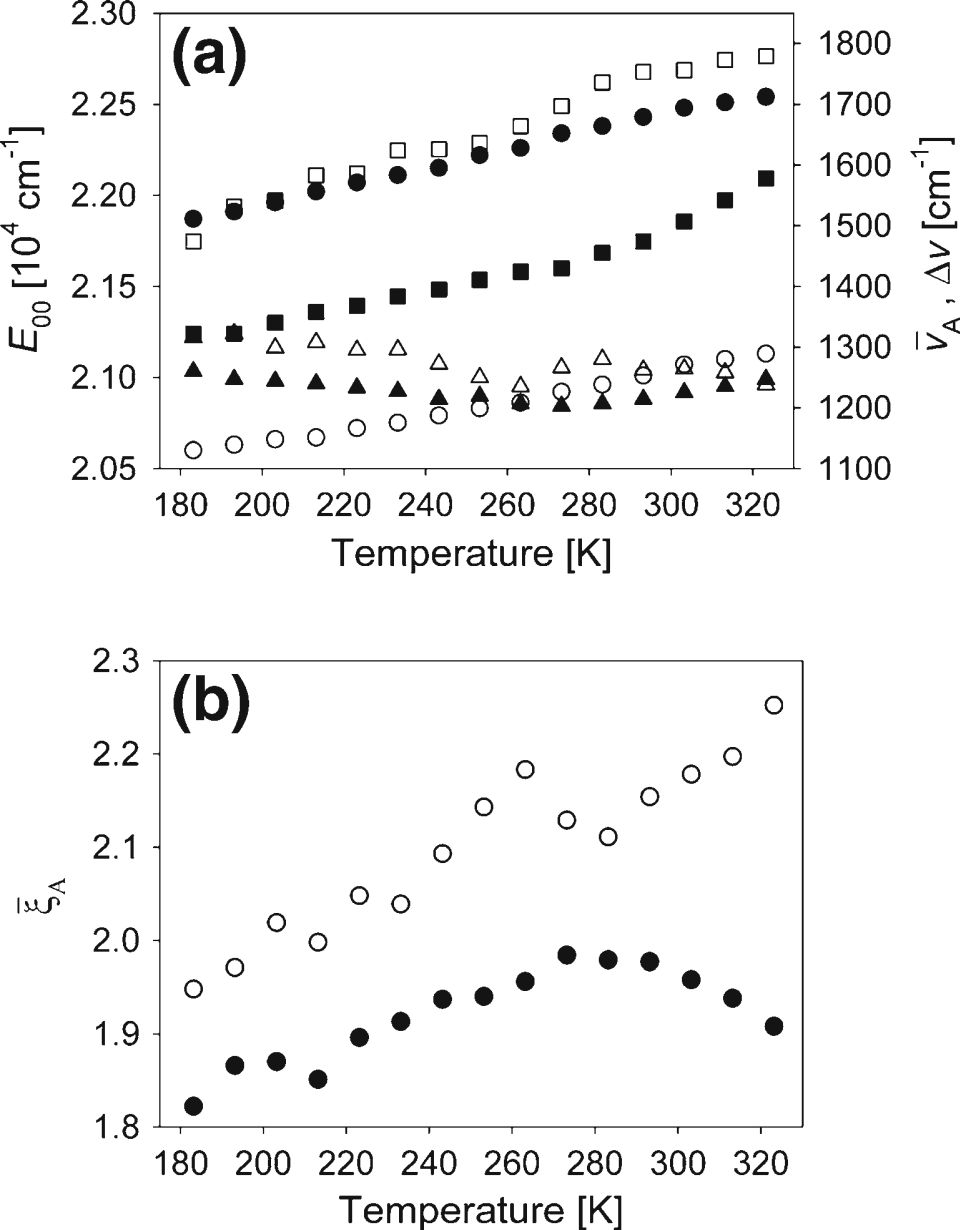
Temperature dependence of: (**a**) *E*_00_, S_1_–S_0_ energy gap for C153 in ClH (*filled circles*) and PPN (*empty circles*), $\Delta {\overline v_{1/2}}$-full width at half-maximum in ClH (*filled squares*) and PPN (*empty squares*), ${\overline v_{\text{A}}}$-in ClH (*filled triangles*) and PPN (*empty triangles*); (**b**) ${\overline \xi_{\text{A}}}$—Huang-Rhys vibrational coupling factor in ClH (*filled circles*) and PPN (*empty circles*) as obtained from fit of C153 emission spectra in both solvents to the model given in Eqs. (4–6)

Similar results were obtained in both solvents, except for *E*_00_ which included a solvent induced shift, thus it was higher in ClH than in PPN (Fig. 10). There is a correlation between ${\overline v_{\text{A}}}$(T), $\Delta {\overline v_{1/2}}$ and ${\overline \xi_{\text{A}}}$(T) dependencies, with significant changes in the same temperature range in which *τ*_F_(T) is observed to change. At this stage it is not possible to deduce how much these correlations affect the results of the fit, thus, to what extent ${\overline v_{\text{A}}}$(T), $\Delta {\overline v_{1/2}}$ and ${\overline \xi_{\text{A}}}$(T) dependencies reflects a true physical effect. These values are averages reflecting changes in different accepting modes properties [22,23]. The similarity between ${\overline v_{\text{A}}}$, $\Delta {\overline v_{1/2}}$ and the corresponding quantities in [13] shows that the 1,150 cm^−1^ (in Fig. 10 ~1,250 cm^−1^) is the dominant mode in the emission process. However, ${\overline \xi_{\text{A}}}$(T) changes are hard to explain on the basis of a single mode deactivation, as they would indicate a rise and then a fall in equilibrium distance with decreasing temperature for C153 in ClH. In PPN an additional preliminary fall in this distance would be expected. However, assuming also that other accepting modes, as the 810 cm^−1^ and 360 cm^−1^ discussed in [7,13], are active in the fluorescence transition a quite simple explanation of the temperature dependence of C153 radiative deactivation can be proposed.

**Fig. 11:**
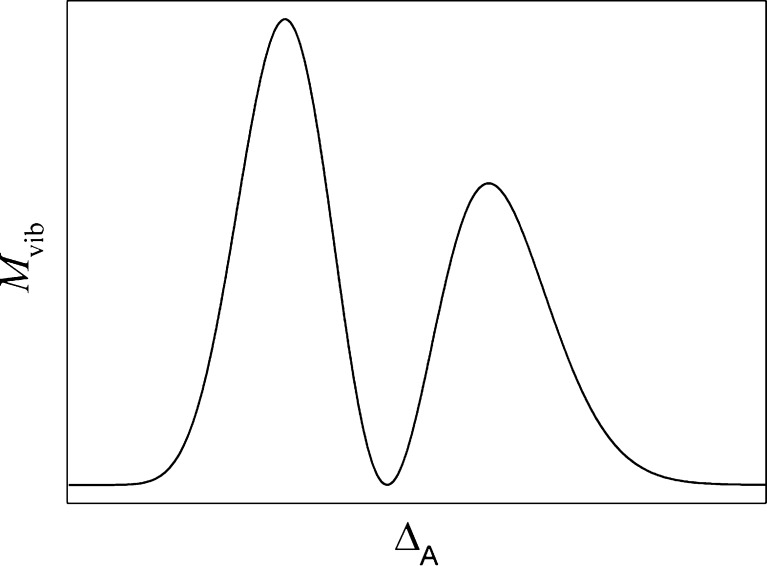
Oscillation with change in equilibrium distance of the Franck-Condon integral of two *S*_0_ and *S*_1_ harmonic oscillator wavefunctions corresponding to the same vibrational mode and two different members of its progression

Both steady-state absorption (Fig. 2) and fluorescence results (Fig. 10) indicate that a change in the equilibrium distance ${\overline \Delta_{\text{A}}}$ occurs on decreasing temperature. According to the Born-Oppenheimer approximation the transition dipole moment between the excited (ψ′′) and ground (ψ′) states is proportional to [24]:

7 $$ {M_{e \to g}} = \left\langle {\psi \prime } \right|\widehat{\mu }\left| {\psi \prime \prime } \right\rangle \propto {P_{el}}\left( {\overline R } \right) \cdot \int {\psi \prime {*_{vib}}\psi \prime {\prime_{vib}}dR}, $$

where ${P_{el}}\left( {\overline R } \right)$ is the purely electronic transition dipole moment, dependent on the *R*-centroid for the transition. The integral represents the overlap of the vibrational wavefunctions in both electronic states. Assuming the harmonicity of the oscillators representing molecular vibrations and no changes in the frequency of a selected oscillator between electronic states involved in the transition, one can quickly check that for a selected vibration mode (frequency) and two selected members of this mode progression in *S*_0_ and *S*_1_ states the integral in Eq. 7 ${M_{vib}} = \int {\psi \prime {*_{vib}}\psi \prime {\prime_{vib}}dR}$ can oscillate with ${\overline \Delta_{\text{A}}}$ changed in the way shown in Fig. 11.

Thus, changes in ${\overline \Delta_{\text{A}}}$ can be the source of similar slight modulation in M_e→g_ for C153 in EtOH and TFEtOH, or in PPN and ClH. Both cases correspond to two different vibrational modes, in alcohols a high frequency one while in PPN and ClH a low frequency one. The modulation in M_e→g_(T) is reflected in the modulations in *τ*_F_(T). Changes in the equilibrium distance must result from changes in the solute-solvent interaction energies.

C153 clearly interacts specifically with protic solvents. Such a conclusion follows from the differences in M_e→g_(T) (Fig. 8) for aprotic and protic solvents and from the values of *τ*_F_(T) obtained for aqueous ClH and PPN compared to the ones obtained for the same solvents when dried properly (Fig. 6). It is also supported by steady-state absorption results. In aqueous ClH and PPN the decrease in T changes the equilibrium between free C153 molecules concentration and C153+H_2_O complexes in favor of the former ones. This is related to the fact water forms clusters of the size increasing with decreasing T [12,25], thus reducing the concentration of water molecules accessible for C153 to form complexes. The difference in M_e→g_ and *k*_nr_ must be small for C153 and its complex with water and we observe an average result as the decay time. This is the case totally different from that of 4-AP which is efficiently quenched by water H-bonding, responsible for the decay times of free 4-AP and 4-AP-water complexes significantly different and separable through optimization routines.

Comparison of C153 M_e→g_ values in protic and aprotic solvents indicates that C153 H-bonding of molecules of protic solvents lead to a change in the equilibrium distance Δ_*A*_ or/and to a slight change in *S*_0_ and *S*_1_ potential energy surfaces shapes. Simultaneously, the steady-state absorption results show that the same interaction in the same solvents leads to an additional stabilization of the $S_1^{FC}$ state with respect to *S*_0_, when compared to what is observed in aprotic solvents. In $S_1^{rel}$ state H-bonds formed by C153 with PPN change in energy which leads to an additional stabilization of the emitting state. We can assume that the same process takes place in EtOH. Such a conclusion follows from Stokes shifts values (∆*v*_S_, Fig. 7). On the other hand, the absorption position temperature dependencies (Fig. 3) and ∆*v*_S_(T) show that in protic TFEtOH the energy of H-bonds formed with C153 decreases with decreasing temperature in the probe $S_1^{rel}$ state, in contrast to what is observed for $S_1^{FC}$. In hydrogen accepting PPN the H-bonds become stronger with decreasing T for C153 in $S_1^{rel}$ state. In EtOH an average result of both processes is observed.

## Conclusions

Overall, we can conclude that the *S*_0_ and *S*_1_ potential energy surfaces undergo two displacements upon changing the solvent and temperature. Horizontal displacement along the equilibrium distance axis induce changes in M_e→g_, *τ*_F_ and ϕ_F_. Vertical displacement in the energy scale changes the absorption and emission positions and *k*_NR_, which thus affects *τ*_F_ and ϕ_F_. Temperature changes imply changes in nonspecific interaction energy. Thus, the induced part of the total dipole moment of the C153 molecule is modified as well. This in turn involves changes in H-bonds energies formed by the probe with TFEtOH, EtOH and PPN in S_0_ and S_1_, of Franck-Condon and relaxed character. At this stage a direct indication of C153 sites/atoms responsible for the experimental effects observed is out of reach. However, on the basis of the steady-state results we can safely conclude that the reorientational relaxation of the solvent accompanying the $S_1^{FC} \to S_1^{rel}$ transition induces a weakening of the H-bonds formed by C153 with protic solvents and a rise in energy of the H-bonds formed with hydrogen accepting solvents. This effects is amplified by a decrease in T and it can be understood as a result of a competition between specific and nonspecific interactions in TFEtOH, and as a result of cooperation between them in PPN. One should keep in mind that in both *S*_1_ states the H-bond energy is higher than in *S*_0_ in protic solvents, while in PPN the H-bond energy in $S_1^{FC}$ is the same as in *S*_0_.
